# Transient acid treatment cannot induce neonatal somatic cells to become pluripotent stem cells

**DOI:** 10.12688/f1000research.4092.1

**Published:** 2014-05-08

**Authors:** Mei Kuen Tang, Lok Man Lo, Wen Ting Shi, Yao Yao, Henry Siu Sum Lee, Kenneth Ka Ho Lee

**Affiliations:** 1 Key Laboratory for Regeneration Medicine, School of Biomedical Sciences, Chinese University of Hong Kong, Shatin, Hong Kong; 2 Faculty of Life Sciences, University of Manchester, Manchester, M13 9PL, UK

## Abstract

Currently, there are genetic- and chemical-based methods for producing pluripotent stem cells from somatic cells, but all of them are extremely inefficient.  However, a simple and efficient technique has recently been reported by Obokata *et al *(2014a, b) that creates pluripotent stem cells through acid-based treatment of somatic cells.  These cells were named stimulus-triggered acquisition of pluripotency (STAP) stem cells. This would be a major game changer in regenerative medicine if the results could be independently replicated. Hence, we isolated CD45 ^+^ splenocytes from five-day-old Oct4-GFP mice and treated the cells with acidified (pH 5.7) Hank’s Balanced Salt Solution (HBSS) for 25 min, using the methods described by Obokata *et al* 2014c. However, we found that this method did not induce the splenocytes to express the stem cell marker Oct4-GFP when observed under a confocal microscope three to six days after acid treatment. qPCR analysis also confirmed that acid treatment did not induce the splenocytes to express the stemness markers *Oct4*, *Sox2* and *Nanog*.  In addition, we obtained similar results from acid-treated Oct4-GFP lung fibroblasts. In summary, we have not been able to produce STAP stem cells from neonatal splenocytes or lung fibroblasts using the acid-based treatment reported by Obokata *et al* (2014a, b, c).

## Introduction

Takahashi and Yamanaka (2006) reported that it was possible to induce adult fibroblasts into pluripotent stem cells using four factors: Oct3/4, Sox2, c-Myc and Klf4. The creation of these induced pluripotent stem (iPS) cells and their replication in human somatic cells has been heralded as a major breakthrough in regenerative medicine ( Takahashi *et al.*, 2007). However, the method for generating iPS cells involves complex genetic manipulation and it is beset by low efficiency of conversion and yield ( Liao *et al.*, 2008). Currently, great strides are being made to improve the efficiency of iPS cell production by incorporating small molecules, vitamin C, valporic acid, microRNAs and different combinations of transcriptional factors into the protocols (reviewed by Huo & Zambidis, 2013; Jung *et al.*, 2014). Two recent sets of groundbreaking studies reported in *Nature* appear to overcome most of these problems ( Obokata *et al.*, 2014a; Obokata *et al.*, 2014b). The authors reported that simply bathing somatic cells in a mild acid could reprogram them to become pluripotent stem cells. They harvested spleen cells from 1-week-old Oct-4-GFP transgenic mice, and isolated the CD45 ^+^ population by flow cytometry. The CD45 ^+^ splenocytes were then treated with acidified HBSS (pH 5.7) for 25 min at 37°C and maintained in DMEM/F-12 culture medium containing B27 and Leukemia Inhibitory Factor (LIF) for one to seven days. This simple procedure activated the Oct4 promoter two days post-treatment, making the splenocytes express the GFP reporter.

The authors named the cells generated by this procedure “Stimulus-triggered acquisition of pluripotency (STAP) stem cells” ( Obokata *et al.*, 2014a). STAP cells were demonstrated to be able to form all organs and tissues in chimeric embryos, when injected into host blastocysts ( Obokata *et al.*, 2014b). Furthermore, the authors reported that STAP cells could also contribute to the placenta, something that iPS and ES cells are normally incapable of doing ( Rossant, 2008). In other words, STAP cells appear to be totipotent rather than pluripotent. If these surprising findings could be confirmed, it would revolutionize regenerative medicine by providing a simple, cheap, and immunocompatible source of stem cells for tissue/organ repair.

There are several fundamental questions that need to be addressed before STAP stem cells can be accepted as one of the main methods for generating stem-like iPS cells, such as: Can these findings be replicated by other researchers? And, other than mouse spleen cells, can this STAP protocol be applied to other somatic cells? Since the publication of the STAP articles, there has been a recent groundswell of comments on social media platforms, including blogs ( Knoeffler Lab Stem Cell Blog), networking sites ( ResearchGate) and Twitter, that have cast considerable doubt on Obokata *et al.*’s findings. In this context, we have attempted to create STAP cells using their most updated protocol ( Obokata *et al.*, 2014c).

## Materials and methods

### Animals

Oct4 *-*GFP transgenic (CBA-Tg (Pou5f1-EGFP) 2Mnn/j) mice, obtained from The Jackson Laboratory and maintained in the CUHK Laboratory Animal Services Centre, were used for experimentation. The usage of these mice was approved by the CUHK Animal Experimentation Ethics Committee (Project No.: 11/056/GRF-5). The new born neonates were kept in a nest bedding with their mothers at 25°C room temperature, under a 12/12 dark-light cycle, and sufficient food and water. We humanely euthanized six to seven of five-day-old neonatal Oct4 *-*GFP mice by cervical dislocation (according to the ARRIVE guidelines, Kilkenny *et al.*, 2012) and harvested their testes, spleens and lungs. This was done on the same day as the mice left animal breeding centre.

### Germ cell isolation and analysis

Testes were harvested from five-day-old neonates and maintained in HBSS medium. The seminiferous tubules were immediately isolated from the testes, under a Nikon SMZ745T stereo dissecting microscope. The tubules were then kept, and maintained in DMEM medium supplemented with 10% qualified Fetal bovine serum (FBS) (Gibco ^®^, Invitrogen; Cat#10270) and penicillin (100U/mL)-streptomycin (0.1mg./mL) (Gibco ^®^, Invitrogen; Cat#15140122). The tubules were examined under a confocal microscope to determine whether the germ cells could express Oct4-GFP. Single germ cell suspensions were also produced from the seminiferous tubules using methods described by ( Garcia & Hofmann, 2012). These germ cells were analyzed using a BD LSRFortessa ^TM^ Cell Analyzer (BD Biosciences, USA) to determine whether these cells were capable of expressing Oct4-GFP.

### Preparation of splenocytes and lung fibroblasts from neonatal mice

Spleens were isolated from five-day-old neonates capable of expressing Oct4 *-*GFP when appropriately induced (e.g. using OSMK factors). The spleens were first mechanically passed through a cell strainer (grid size 70µm) to disperse the tissues and dissociate the splenocytes. The splenocytes were pelleted by centrifugation at 1200 rpm for five min and resuspended in ACK lysis buffer (65mM NH _4_Cl, 10mM KHCO _3_ and 0.1M Na _2_-EDTA in distilled H _2_O, adjusted to pH 7.3) for five min at room temperature to remove residual erythrocytes. The splenocytes were then resuspended in DMEM supplemented with 10% FBS, and 1% PS. The crude splenocytes were maintained at 37°C and 5% CO _2_ for one to six days.

Besides splenocytes, we also isolated fibroblasts from the lungs of the Oct4 *-*GFP neonates ( Yau *et al.*, 2011). Briefly, explants were prepared from dissected pieces of the lung (approximately 1 mm ^2^ in size). The explants were treated with 1 mg/ml collagenase Type I (Gibco ^®^, Invitrogen) for 30 min. The explants were then washed with PBS and plated onto collagen-coated 100 mm culture dishes (SPL; Cat#20101). DMEM/F12 plus 10% FBS were added to the explants (just enough to cover the explants). These explants were then maintained at 37°C and 5% CO _2_, with the culture medium changed every three days. Fibroblasts could be observed migrating out of the explants after two days. After seven days, the explants were removed and the fibroblasts on the culture dishes were trypsinized with 0.25% Trypsin-EDTA (Gibco ^®^, Invitrogen) for two minutes and sub-cultured.

### Isolation of CD45 ^+^ splenocytes

The crude splenocytes produced above were resuspended in PBS into a single-cell (1 × 10 ^7^ cells/ml) suspension. The cells were incubated with Anti-mouse CD45 antibodies, which were directly conjugated with FITC (1: 100 dilutions, BD Pharmingen ^TM^ FITC Rat Anti-mouse CD45) for 30 min at 4°C. The stained cells were then washed three times with PBS and resuspended in PBS supplemented with 1% FBS. The CD45 ^+^-stained splenocytes were sorted and purified using a Cell Sorter (BD LSRFortessa Cell Analyzer). The cells were sorting on 3 different occasions using the same pool of splenocytes extracted from the spleens of 6 neonates.

### Acid treatment of CD45 ^+^ splenocytes

We treated the somatic cells with low-pH medium according to the protocol described in ( Obokata *et al.*, 2014c). Accordingly, 5×10 ^5^ cells/ml of CD45 ^+^ splenocytes or lung fibroblasts were treated with low-pH HBSS (adjusted to pH 5.7 with HCl) for 25 min at 37°C. The acid-treated cells were then centrifuged at 1200 rpm for 5 min. The supernatants were removed and rechecked to confirm that the pH was still pH 5.7. All pH were measured using a calibrated pH meter (Mettler-Toledo, USA). All of acid-treated cell pellets were resuspended in DMEM/F-12 medium supplemented with 1× B27 and 1,000U LIF at a concentration of 1×10 ^5^ cells/ml. The cultures were then plated onto non-adhesive culture dishes (SPL; Cat#11035) and examined for GFP expression on seven consecutive days using microscopy (LEICA SP5). The experiments were performed in triplicate.

### Real-Time quantitative PCR analysis

The acid-treated and untreated cells were harvested for qPCR after six-seven days of culture. Briefly, total RNAs were isolated using an RNeasy ^^®^^ mini kit (Qiagen, USA) and reverse transcribed using an Omniscript RT Kit (Qiagen, USA). We used National Institutes of Health’s qPrimer Depot Database to determine the primers for *Oct4* (forward: 5′GTTGGAGAAGGTGGAACCAA3′; reverse: 5′TCTTCTGCTTCAGCA GCTTG3′), *Sox2* (Forward: 5′ACAAGAGAATTGGGAGGGGT3′; reverse: 5′AAAGCGTTAATTTGGATGGG3′) and *Nanog* (forward: 5′CCAGTGGAGT ATCCCAGCAT3′; reverse: 5′GAAGTTATGGAGCGGAG CAG3′) expressions. The master mix for each qPCR sample was prepared to a total volume of 5 µl for a 384 well-plate: 10 ng cDNA template, 0.55 µM forward primer, 0.55 µM reverse primer, SYBR green master premix (Takara Biotechnology Co Ltd, Dalian) and RNAse-free water (Ambion, Cat#AM9937). The qPCR was performed with a denaturation step at 95°C for 30 sec and thermal profiling (denaturation step: 95°C, 5 sec; annealing and extension steps: 42°C, 30 sec) for 40 cycles (ABI ViiA 7 Real Time PCR System). After the process was completed, the dissociation and amplification curves were checked to see if there was any abnormal amplification. The Ct values were further measured and acquired. The data were generated for quantitative analyses after normalization with GAPDH housekeeping gene. All samples were run in triplicate.

### Statistics

The data were analyzed using two-tailed, paired student’s t-test. P<0.05 was considered to be statistically significant. The statistical analysis was performed using SPSS software version 22.

## Results

### Validation of Oct4-GFP transgenic mice

**Figure 1.**
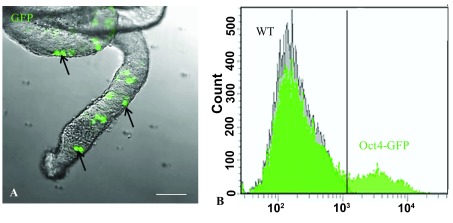
Analysis of germ cells from the testes of five-day-old Oct4-GFP neonates. ( **A**) Confocal image of seminiferous tubules isolated from five-day-old neonates showing the germ cells expressing Oct4-GFP (arrows). Bar = 50µm. ( **B**) Flow cytometry histogram confirming that the germ cells are capable of expressing the transgene. The peaks show that only Oct4-GFP germ cells but not wild type (WT) germ cells expressed GFP.

We first confirmed that the cells in our Oct4-GFP transgenic mice were capable of expressing Oct4-GFP. It has been reported that all of the spermatogonia in the testes of this type of transgenic mice were capable of expressing Oct4 ( Denn *et al.*, 2008). Hence, we isolated the seminiferous tubules from the testes of our transgenic mice and directly examined the tubules under a confocal microscope. We determined that there were numerous Oct4-GFP ^+^ spermatogonia present in the tubules ( Figure 1A). This was further validated by flow cytometery of dissociated germ cells extracted from the seminiferous tubules ( Figure 1B).

### Effects of acid treatment on the neonatal Oct4-GFP splenocytes and lung fibroblasts

**Figure 2.**
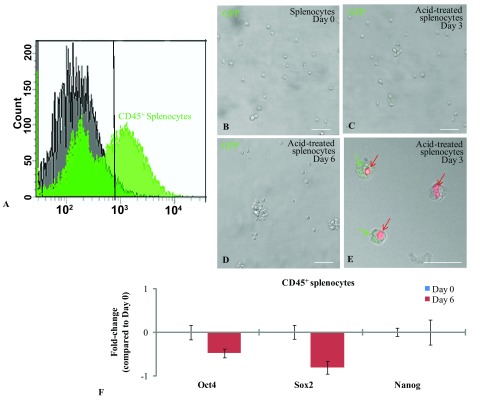
Induction of Oct4-GFP expression in acid-treated splenocytes. ( **A**) Flow cytometry histogram showing the sorted CD45 ^+^ splenocytes used in our experiments. ( **B**– **E**) Confocal images of acid-treated splenocytes after zero to six days of culture. No Oct4-GFP expression was detected in the splenocytes at any of the time-points analyzed. Bar = 50µm. ( **E**) Confocal image of acid-treated splenocytes stained with Propidium iodide (PI) after three days of culture. The PI dye identified the necrotic splenocytes (red arrows) which occasionally also emitted a green autoflorescence (green arrows). Bar = 25µm. ( **F**) qPCR analysis also confirmed that acid-treatment did not induce the splenocytes to express the stemness markers *Oct4*, *Sox2* and *Nanog*. Error bars represent standard error of the mean (p≤0.05).

To replicate Obokata’s experiment, we isolated a pure population of CD45 ^+^ splenocytes from the spleens of five-day-old Oct4-GFP mice as shown in Figure 2A. The CD45 ^+^ splenocytes were treated with a mild acidic (pH 5.7) HBSS for 25 min at 37°C. The acid-bathed cells were then cultured in DMEM/F-12 medium supplemented with B27 and 1,000U LIF. We examined the cells for Oct4-GFP expression, every day for up to seven days, but did not observe any Oct4-GFP expression under the confocal microscope ( Figures 2B, C and D). However, we did occasionally observe clusters of cells that appeared to be GFP ^+^ but were later determined to be autofluorescence, as these cells were necrotic and stained positive with propidium iodide ( Figure 2E). qPCR analysis was performed to establish whether the acid-bathed cells were capable of expressing the stemness markers. The qPCR results revealed that acid treatment did not induce the splenocytes to express *Oct4*, *Sox2* and *Nanog* ( Figure 2F).

**Figure 3.**
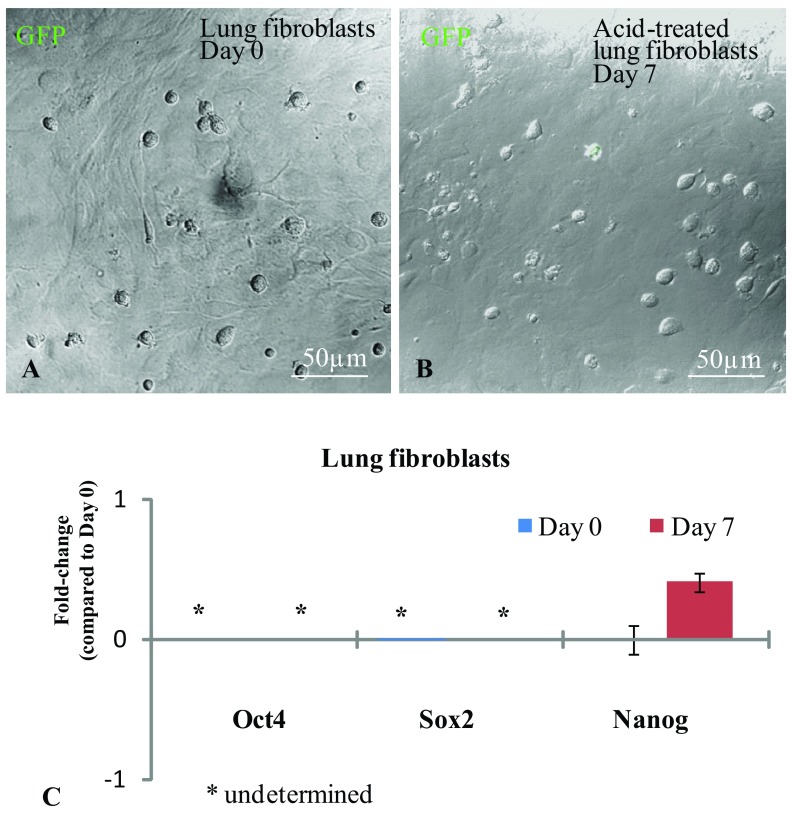
Induction of Oct4-GFP expression in acid-treated lung fibroblasts. ( **A** and **B**) Confocal images of acid-treated fibroblasts zero and seven days after culture. None of the acid-treated fibroblasts expressed the Oct4-GFP transgene. ( **C**) qPCR analysis demonstrated that acid-treatment did not induce *Oct4*, *Sox2* and *Nanog* expression. No expression was determined after 40 cycles of qPCR for *Oct4* and *Sox2*, hence marked as ‘undetermined’. Error bars represent standard error of the mean (p≤0.05).

We also attempted to produce STAP cells from Oct4-GFP lung fibroblasts. Like the splenocytes, the fibroblasts were treated with acidic HBSS (pH 5.7) for 25 min at 37°C. We checked the cells for GFP expression for seven consecutive days but did not observe any GFP expression ( Figure 3A and B). Likewise, our qPCR analysis also did not show induced *Oct4*, *Sox2* and *Nanog* expressions in our acid-treated fibroblasts ( Figure 3C).

Dataset 1 and 2. qPCR results of CD45+ splenocytes/ lung fibroblasts. qPCR data were generated using Viia7 real-time PCR system software. Ct: Threshold cycle number where florescence signal of each sample reached threshold level as defined by the software. Undetermined: Florescence signal of the samples did not reach the threshold level before maximum (40) PCR cycles of each run. SD: Standard deviation of Ct of technical triplicate of each biological sample.

## Discussion

Currently, there is a trend to simplify iPS cell production by minimizing genetic manipulation and incorporating the use of small chemical molecules for somatic cell reprogramming ( Shi *et al.*, 2008; Zhu *et al.*, 2010). In this context, it has been reported that mouse iPS cells could be generated using a cocktail of seven chemical molecules without any genetic manipulation, with an efficiency of around 0.2% ( Hou *et al.*, 2013). These important developments were recently superseded by claims that hydrochloric acid treatment alone can chemically reprogram fibroblasts to become induced pluripotent stem cells ( Obokata *et al.*, 2014a; Obokata *et al.*, 2014b). The authors claimed that chemically stressing CD45 ^+^ splenocytes (isolated from Oct4-GFP neonates) to the point of death so that approximately 25% of the cells survive will activate the Oct4-GFP transgene in over 50% of surviving cells. They called these cells “STAP cells” which, when injected into host blastocysts, could participate in the development of all tissues and organs, including the placenta. The STAP chimeric mice produced were reported to be healthy, and the STAP-derived germ cells were demonstrated to be involved in germline transmission ( Obokata *et al.*, 2014a; Obokata *et al.*, 2014b). These are astonishing findings. Nevertheless, we have tried to replicate the first stages of Obokata’s findings using CD45 ^+^ splenocytes isolated from Oct4-GFP neonates, but could not activate the expression of the Oct4-GFP transgene. This is despite using their most updated protocol for producing STAP cells, which was reported in *Protocol Exchange* ( Obokata *et al.*, 2014c). We also tried using Oct4-GFP lung fibroblasts instead of splenocytes, but again we failed to detect Oct4-GFP expression after acid-treatment. Occasionally, there were cells that appeared GFP positive, but we later determined them to be autofluorescence from apoptotic cells. We made sure that the pH was exactly maintained at pH 5.7 during the experiments by measuring the pH before and after cell treatment. This is because the *Protocol Exchange* protocol placed a lot of emphasis on maintaining an optimal pH during the acid treatment of the cells. We found that there was a pH 0.1 increase after the acid buffer was added to treat the cells – so our starting pH was actually 5.6 to compensate. At the end of acid bath stimulation, we also measured the pH of the buffer to confirm that it was still pH 5.7. Therefore, our inability to produce STAP cells could not be attributed to changes in the pH during the cell stimulus procedures.

Another possibility why we could not replicate Obokata’s results might be the difference in the strains of Oct4-GFP transgenic mice used. We acquired our transgenic mice from The Jackson Laboratory (CBA-Tg (Pou5f1-EGFP) 2Mnn/j) while Obokata used transgenic mice generated by Ohbo *et al.*, 2003. Their transgenic mice were developed from a C57BL.6J background, and carry the EGFP cDNA under the control of an Oct4 18-kb genomic fragment (consisting of a minimal promoter and proximal and distal enhancer). Perhaps the transgene in these mice is more easily activated than in our Jackson Laboratory mice. This could potentially explain why Obokata’s transgenic splenocytes, but not our transgenic splenocytes, expressed the EGFP reporter following acid bath treatment. Nevertheless, in the context of generating STAP stem cells, it is not the expression of the transgene that is important but rather the expression of the endogenous Oct4 gene - and related endogenous stemness genes, Sox2 and Nanog. Expression of these genes could not be demonstrated using qPCR analysis following splenocyte acid treatment and culture.

In conclusion, we have not been able to replicate Obokata *et al.*’s findings to produce STAP stem cells from somatic cells. It appears that the method for producing STAP stem cells is not as simple and straight forward as has been reported.

## Data availability

*figshare:* Dataset 1 and 2. qPCR results of CD45+ splenocytes/lung fibroblasts. Doi: 10.6084/m9.figshare.1014318 ( Tang *et al.*, 2014)
