# Supplementary material for: Transient acid treatment cannot induce neonatal somatic cells to become pluripotent stem cells
Source: F1000Res. 2014 May 8;3:102. [Version 1] doi: 10.12688/f1000research.4092.1 (PMC4032108; doi:10.12688/f1000research.4092.1)
Supplement: Peer review history

# Peer review: Referee response for version 1

**DOI:** 10.5256/f1000research.4382.r4845

**Published:** 2014-05-21

**Authors:** Janet Rossant

**Affiliations:** 1 Developmental and Stem Cell Biology, Hospital for Sick Children, Toronto, ON, Canada

Tang *et al.* report attempts to replicate the findings of *Obokata et al.* on the derivation of pluripotent STAP cells by acid treatment of mouse splenocytes. Despite attempting to replicate the exact details of the Obokata experiments as published, they were not able to observe any activation of an Oct4-GFP transgene or of the endogenous pluripotent markers after several days of culture of the treated cells.

While these results do suggest that the STAP protocol is not simple and easily replicated, negative findings are always hard to interpret and need to be very carefully controlled. For this reason there are some points that need to be considered:

1.  It is not clear how much cell death occurred after the acid treatment - was this at the same level as reported by *Obokata et al.*? 
2. What was the sensitivity of the PCR analysis of the expression of the pluripotent genes? A control analysis of different levels of expression in existing pluripotent lines would help.
3.  Is it possible to repeat the analysis with a different Oct4-reporter to avoid the potential confusion with green autofluorescence?

There is merit in this replication study being published in the scientific literature, however when trying to reproduce a null finding the controls used need to be well defined to prevent any ambiguity.

I have read this submission. I believe that I have an appropriate level of expertise to confirm that it is of an acceptable scientific standard.

# Response

**Published:** 2014-05-24

**Authors:** Kenneth Lee

**Affiliations:** School of Biomedical Sciences, Chinese University of Hong Kong, Hong Kong

Professor Rossant has made some excellent suggestions for improving the paper.

Points 2 and 3 will be address by adding positive and negative controls into a revised version of the paper.  This will demonstrate the sensitively of our Oct4-GFP transgene. qPCR data will also accompany these results which will address the sensitivity of the PCR analysis question. To address point 1, how “much cell death occurred after the acid treatment”, we are currently checking over all our stored confocal images that have been stained with PI dye to derive an estimate.  We will try and integrate this data into the revised version of the paper.  

To date, we have already generated a set of negative control results but the positive control will take some time to generate. We will do it by chemical induction of our Oct4-GFP lung fibroblasts using a cocktail of small molecules. We will try and have a revised version of the paper uploaded as soon as possible.

# Peer review: Referee response for version 1

**DOI:** 10.5256/f1000research.4382.r4727

**Published:** 2014-05-09

**Authors:** Christine Mummery

**Affiliations:** 1 Department of Anatomy and Embryology, Leiden University Medical Center, Leiden, Netherlands

The authors describe their attempt to reproduce a study in which it was claimed that mild acid treatment was sufficient to reprogramme postnatal splenocytes from a mouse expressing GFP in the oct4 locus to pluripotent stem cells. The authors followed a protocol that has recently become available as a technical update of the original publication.

They report obtaining no pluripotent stem cells expressing GFP driven over the same time period of several days described in the original publication. They describe observation of some green fluorescence that they attributed to autofluorescence rather than GFP since it coincided with PI positive dead cells. They confirmed the absence of oct4 expression by RT-PCR and also found no evidence for Nanog or Sox2, also markers of pluripotent stem cells.

The paper appears to be an authentic attempt to reproduce the original study, although the study might have had additional value with more controls: “failure to reproduce” studies need to be particularly well controlled.

Examples that could have been valuable to include are:

1. For the claim of autofluorescence: the emission spectrum of the samples would likely have shown a broad spectrum not coincident with that of GFP.
2. The reprogramming efficiency of postnatal mouse splenocytes using more conventional methods in the hands of the authors would have been useful as a comparison. Idem the lung fibroblasts.
3. There are no positive control samples (conventional mESC or miPSC) in the qPCR experiments for pluripotency markers. This would have indicated the biological sensitivity of the assay.
4. Although perhaps a sensitive issue, it might have been helpful if the authors had been able to obtain samples of cells (or their mRNA) from the original authors for simultaneous analysis.

 In summary, this is a useful study as it is citable and confirms previous blog reports, but it could have been improved by more controls.

I have read this submission. I believe that I have an appropriate level of expertise to confirm that it is of an acceptable scientific standard.

# Response

**Published:** 2014-05-12

**Authors:** Kenneth Lee

**Affiliations:** School of Biomedical Sciences, Chinese University of Hong Kong, Hong Kong

Professor Mummery has provided some excellent suggestions for changes to improve the paper.  We will try our best and accommodate her requests 1-3 by doing some new additional experiments.

Request 4 will be next to impossible to address, I don’t think Haruko Obakata will be willing to give us her STAP stem cells- even if she does it will be problematic for us to establish they were originally derived from mouse splenocytes.
